# The Role of Movement Patterns in Epidemic Models on Complex Networks

**DOI:** 10.1007/s11538-021-00929-w

**Published:** 2021-08-19

**Authors:** Alfonso Ruiz-Herrera, Pedro J. Torres

**Affiliations:** 1 Department of Mathematics, Faculty of Science, University of Oviedo, Oviedo, Spain; 2 Department of Applied Mathematics, Faculty of Science, University of Granada, Granada, Spain

**Keywords:** Optimal topology, Degree of mobility, Management guidelines, Patchy models

## Abstract

In this paper, we analyze the influence of the usual movement variables on the spread of an epidemic. Specifically, given two spatial topologies, we can deduce which topology produces less infected individuals. In particular, we determine the topology that minimizes the overall number of infected individuals. It is worth noting that we do not assume any of the common simplifying assumptions in network theory such as all the links have the same diffusion rate or the movement of the individuals is symmetric. Our main conclusion is that the degree of mobility of the population plays a critical role in the spread of a disease. Finally, we derive theoretical insights to management of epidemics.

**Supplementary Information:**

The online version supplementary material available at 10.1007/s11538-021-00929-w.

## Introduction

The spatial structure and the mobility of a population are critical factors for the control of any epidemic (Danon 2011; Heesterbeek 2015; Khan 2012; Prabodanie et al. 2020). In today’s world, with communities more connected than ever, the total eradication of an epidemic requires joint and coordinated efforts across different cities and countries. Contact tracing, quarantine or vaccination programs are some examples of control strategies that generally involve variables related to the movement of individuals (Heesterbeek 2015; Silk 2019; Tien and Earn 2010; Keeling and Rohani 2011). Yet even though these strategies are a possible solution in many cases, our knowledge on their precise effect is still under development.

Mathematical models offer a valuable tool to study these challenges. A natural manner to study the spread of an epidemic is to divide the location of the population into discrete subpopulations and consider a metapopulation. In the recent years, there has been an increased interest in metapopulation models with regard to the existence and stability of the disease free and endemic equilibria (Allen et al. 2007; Arino et al. 2016, 2012; Arino 2009; Artzy-Randrup and Stone 2010; Castillo-Chavez et al. 2016). Generally speaking, the basic reproduction number of the metapopulation $\mathcal {R}_{0}$ determines the uniform persistence/global stability of the disease free equilibrium. These results normally indicate that the network topology plays a paramount role in the dynamics of the epidemic. However, there are many unsolved questions in network theory. To the date, the majority of the works in spatial epidemiology assumes some type of simplifying condition such as all the links have the same diffusion rate or the movement of the individuals is symmetric (see Artzy-Randrup and Stone 2010; Lamouroux et al. 2015; Keeling and Eames 2005 and the references therein). Although these assumptions allow the treatment of some complex models, they normally lead to misleading conclusions in real-world problems. For example, the condition that all patches are equally accessible for dispersers generally produces a fictitious homogenization of the population.

In this paper we develop a new methodology to study the influence of the spatial topology on the total number of infected individuals. Specifically, given two spatial topologies, we are able to deduce which topology leads to less infected individuals. In particular, we determine the topology that minimizes the overall number of infected individuals. In our analysis, we study the classical SIR model for a spatially distributed population where patches are interconnected in any fashion. Importantly, we do not assume any of the simplifying assumptions mentioned above. From a theoretical point of view, the motivation is to understand the interplay between the different movement patterns and the common epidemic variables. On the other hand, implementing a management practice requires a previous analysis of the expected risks and costs. From a more applied side, our paper suggests several theoretical recommendations in control strategies related to the spatial topology. For instance, we have in mind the questions: What connections should we remove to reduce the number of infected individuals? When does the movement of individuals between two cities increase the number of infected individuals?

## Material and Methods

We consider a population distributed in *n* different patches. Each subpopulation is divided into three subgroups: susceptible ($S_{i}(t)$), infected ($I_{i}(t)$) and recovered ($R_{i}(t)$) individuals. Susceptible individuals contract the disease with a rate $\beta _{i}$, only through the contact with infected individuals that inhabit the same patch. The recovery and birth rates are denoted by $\gamma _{i}$ and $\lambda _{i}$, respectively. All newborns are susceptible. The individuals die with rate $\mu _{i}$ independently of their current state. For simplicity, we assume that the mortality rate does not depend on the patch, i.e., $\mu _{i}=\mu$ for all $i=1,\ldots ,n$. The classical SIR metapopulation model with patch structure (see Allen et al. 2007; Castillo-Chavez et al. 2016; Lamouroux et al. 2015) is

1 $$\begin{aligned} \left\{ \begin{array}{lll} S_{i}' &{}=&{} \lambda _{i}-\mu S_{i}-\beta _{i} S_{i} I_{i}+\sum _{j=1}^{n}A_{ij} S_{j}\\[1ex] I_{i}' &{}=&{} \beta _{i} S_{i} I_{i}-(\gamma _{i} +\mu )I_{i}+\sum _{j=1}^{n}B_{ij} I_{j}\\[1ex] R_{i}' &{}=&{} \gamma _{i} I_{i}-\mu R_{i}+\sum _{j=1}^{n}C_{ij} R_{j}\;\;\;\;\;\;\;\;\;\;\;\;{\mathrm{for}}\;\; i=1,\ldots ,n \end{array}\right. \end{aligned}$$

with $A_{ij}$, $B_{ij}$ and $C_{ij}$ nonnegative diffusion coefficients for the susceptible, infected, and recovered individuals respectively. We write $A_{ij}=a_{ij}h$ where *h* represents the average number of changes of patch of an individual per unit of time and $a_{ij}\in [0,1]$ is the probability that an individual from patch *j* uses a route ending at the patch *i* (with $i\not =j$). If $h=0$, the individuals never change their location. However, a large value of *h* indicates an ample tendency to move. On the other hand, $a_{ij}=0$ means that the susceptible individuals cannot use the route from patch *j* to patch *i*, whereas a large value of $a_{ij}>0$ implies that the route is very likely to be used. Analogously, we write $B_{ij}=b_{ij}h$ and $C_{ij}=c_{ij}h$. We assume that there are no births and deaths during travel. Thus,

2 $$\begin{aligned} \sum _{i=1}^{n}a_{ij}=\sum _{i=1}^{n}b_{ij}=\sum _{i=1}^{n}c_{ij}=0 \end{aligned}$$

for all $j=1,\ldots ,n$ with

3 $$\begin{aligned} a_{ii}\le 0, b_{ii}\le 0, c_{ii}\le 0\;\;\;and\;\;\;a_{ij}, b_{ij},c_{ij}\ge 0. \end{aligned}$$

If both subindices coincide, i.e., $a_{ii},b_{ii},c_{ii}$, they do not represent probabilities. Actually, for example, $$1+a_{ii}=1-\sum \nolimits _{{\begin{array}{c} j=1\\ j\not =i \end{array}}}^{n}a_{ji}$$ is the probability that a susceptible individual of patch *i* remains in that patch (Arino 2009; Arino et al. 2016). In model (1), we study a heterogeneous metapopulation. In particular, the patches sizes are in general different each other. Although we do not introduce an specific parameter for it, any epidemic parameter in (1) depends critically on the patch size.

For the analysis of model (1), we employ a graph for each subgroup of individuals. Network theory has experienced a considerable growth in different fields in last years. It is rather common to find different definitions to refer to the same notion depending on the field. To avoid misleading conclusions in the literature, we fix the definitions employed in this paper. The network topology (also known as network architecture) for the susceptible individuals refers to the collection of all the available routes for these individuals. In model (1), there is an infinite number of matrices associated with the same network topology. Specifically, two matrices $\mathcal {A}=(a_{ij})$ and $\widetilde{\mathcal {A}}=(\widetilde{a}_{i j})$ that satisfy (2) and (3) represent the same network topology if

$$\begin{aligned} a_{ij}>0 \Longleftrightarrow \widetilde{a}_{ij}>0 \end{aligned}$$

for all $i,j=1,\ldots ,n$ with $i\not =j$. The matrix $\mathcal {A}=(a_{ij})$ is often coined as connectivity matrix. According to Artzy-Randrup and Stone (2010), the adjacency matrix is easily reconstructed from the connectivity matrix by setting diagonal entries to zero. A network topology for the susceptible individuals is symmetric when $a_{ij}>0$ implies that $a_{ji}>0$. In other words, if there exists a link from patch *j* to patch *i*, then there exists a link from patch *i* to patch *j* as well. Finally, the movement of the susceptible individuals is symmetric if $a_{ij}=a_{ji}$, that is, an individual moves from patch *i* to patch *j* with the same probability as from patch *j* to patch *i*. Obviously, a symmetric movement implies a symmetric topology but not vice versa. We employ the analogous notions for the infected and recovered individuals. A square matrix of order *n* is irreducible if for each pair of indices $i,j\le n$, there is a sequence of indices $i_{1},\ldots ,i_{k}$ so that $a_{i i_{1}}>0$, $a_{i_{1}i_{2}}>0$,..., $a_{i_{k}j}>0$. From a biological perspective, irreducibility means that any two different patches can be always connected by a sequence of paths.

## Results

Our aim is to describe the network topologies that minimize the total population size of infected individuals at equilibrium

4 $$\begin{aligned} T_{I}(h)=\sum _{i=1}^{n} I_{i}^*(h), \end{aligned}$$

where $(S^*_{1}(h),I^*_{1}(h),R^*_{1}(h),\ldots ,S^*_{n}(h),I^*_{n}(h),R^*_{n}(h))$ is the equilibrium of system (1), which is a global attractor. If we consider $T_I(h)$ as a function of *h*, the derivative $T_I'(h)$ with respect to *h* provides information about the sensitivity of $T_I(h)$ with respect to small variations of the mobility parameter *h*. We distinguish three cases: populations with a reduced mobility ($h\approx 0$), populations with a high degree of mobility ($h\approx +\infty$) and intermediate degrees of mobility. In the first case, since $T_{I}(0)$ is independent of the matrices $\mathcal {A}$, $\mathcal {B}$ and $\mathcal {C}$, we can minimize $T_{I}(h)$ by finding the topology that minimizes $T_{I}'(0)$. Hence, we aim to minimize $T'_{I}(0)$ provided the connectivity matrices $\mathcal {A}=(a_{ij})$, $\mathcal {B}=(b_{ij})$ and $\mathcal {C}=(c_{ij})$ satisfy conditions (2) and (3). In the second case, we minimize

$$\begin{aligned} \lim _{h\rightarrow +\infty }T_{I}(h). \end{aligned}$$

A critical task in real problems will be to determine when a particular value of *h* is a small parameter. To overcome this, we have to compare *h* with the smallest parameters of (1).

Many papers in epidemiology focus on a reduced number of topologies for the spatial network. Some popular options are the fully connected network, where direct dispersal from every patch to every patch is possible, or the Erdös–Rényi random graphs (Artzy-Randrup and Stone 2010). We stress that our main aim is to find the graphs or topologies that minimize the total number of infected individuals. Therefore, we do not impose any restriction on the type of graphs employed in this paper.

### Optimal Topologies for Populations with Reduced Mobility in Model (1)

The basic reproduction number within patch *i* in the absence of movement is

$$\begin{aligned} \mathcal {R}_{0,i}=\frac{\beta _{i}\lambda _{i}}{\mu (\gamma _{i}+\mu )}. \end{aligned}$$

This quantity determines the dynamical behavior of the system

5 $$\begin{aligned} \left\{ \begin{array}{lll} S_{i}' &{}=&{} \lambda _{i}-\mu S_{i}-\beta _{i} S_{i} I_{i}\\[1ex] I_{i}' &{}=&{} \beta _{i} S_{i} I_{i}-(\gamma _{i} +\mu )I_{i}\\[1ex] R_{i}' &{}=&{} \gamma _{i} I_{i}-\mu R_{i}. \end{array}\right. \end{aligned}$$

Concretely, if $\mathcal {R}_{0,i}>1$, there is an endemic equilibrium that is a global attractor. Otherwise, the disease is eradicated. The next discussion critically depends on $\mathcal {R}_{0,i}$ for all $i=1,\ldots ,n$.

#### The Disease is Endemic in All Patches ($\mathcal {R}_{0,i}>1$ for $i=1,\ldots ,n$)

We have derived in Electronic Supplementary Material (ESM) that

6 $$\begin{aligned} T_{I}'(0)=\sum _{{\begin{array}{c} i,j=1\\ i\not =j \end{array}}}^{n}a_{ij}\frac{1}{\beta _{j}}\left( \frac{\Delta _{j}}{\Delta _{i}}-1\right) +\sum _{{\begin{array}{c} i,j=1\\ i\not =j \end{array}}}^{n}b_{ij}\frac{\mathcal {R}_{0,j}\mu }{\beta _{j}\Delta _{j}} \left( \frac{\Gamma _{i}}{\Gamma _{j}}-1\right) \end{aligned}$$

with $$\Delta _{i}=\gamma _{i}+\mu $$ and

7 $$\begin{aligned} \Gamma _{i}=\frac{\mathcal {R}_{0,i}}{\Delta _{i}(\mathcal {R}_{0,i}-1)}. \end{aligned}$$

Since there is no reinfection of recovered individuals, the diffusion parameters $c_{ij}$ of the recovered individuals do not appear in (6). Another observation is that there are no additional benefits of the network itself, just independent effects of all the routes. For populations with reduced mobility, if two different topologies produce the same value of (6), then the total number of infected individuals behaves in the same manner (for small values of *h*).

The contribution of the movement of susceptible individuals in the route from patch *j* to patch *i* in formula (6) is

8 $$\begin{aligned} a_{ij}\frac{1}{\beta _{j}}\left( \frac{\Delta _{j}}{\Delta _{i}}-1\right) \end{aligned}$$

If $\Delta _{j}<\Delta _{i}$ or equivalently, the recovery rates satisfy $\gamma _{j}<\gamma _{i}$, then the movement of the susceptible individuals from patch *j* to patch *i* reduces the number of infected individuals and the movement in the opposite sense increases it. Thus, the recommendation to reduce the number of infected individuals is to use a graph for the susceptible individuals with only routes in which the recovery rates of the departing patches are smaller than those of the arriving patches. Following this rule of thumbs, we obtain a directed graph with the arrows always pointing to the patch with the largest value of the recovery rate ($\gamma$).

The contribution of the movement of infected individuals in the route from patch *j* to patch *i* in formula (6) is

9 $$\begin{aligned} b_{ij}\frac{\mathcal {R}_{0,j}\mu }{\beta _{j}\Delta _{j}} \left( \frac{\Gamma _{i}}{\Gamma _{j}}-1\right) . \end{aligned}$$

The analysis of the benefits/damages of this route is analogous to that of the same route for the movement of the susceptible individuals just replacing $\Delta _{i}$ by $\Gamma _{j}$. Specifically, if $\Gamma _{i}<\Gamma _{j}$, the movement of the infected individuals from patch *j* to patch *i* reduces the number of infected individuals. By expression (7), $\Gamma _{i}$ is smaller than $\Gamma _{j}$ when the patch *j* is close to becoming disease-free. As above, the topology for the infected individuals that minimizes the number of infected individuals is a directed graph with the arrows always pointing to the region with the smallest value of $\Gamma$.

**Fig. 1:**
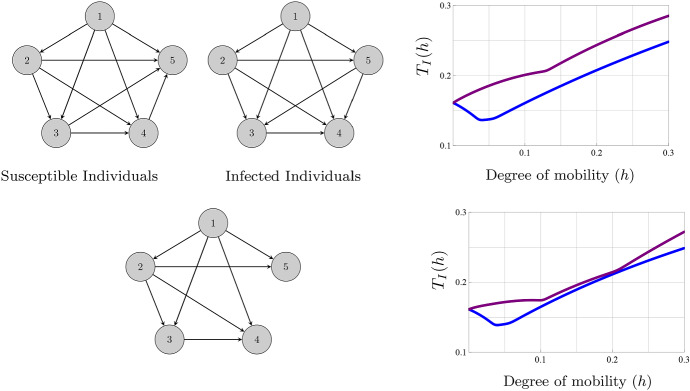
(Color figure online) Optimal topologies for the susceptible and infected individuals and representation of the total number of infected individuals $T_{I}(h)$ (blue curves) depending on the degree of mobility of the population ($h\in (0,0.3)$). The network is made of five patches with parameters $\lambda _{i}=0.5$, $\mu =0.5$, $\beta _{1}=1.3$, $\beta _{2}=1.5$, $\beta _{3}=1.9$, $\beta _{4}=2$, $\beta _{5}=2.1$, $\gamma _{1}=0.7$, $\gamma _{2}=0.9$, $\gamma _{3}=1.1$, $\gamma _{4}=1.2$ and $\gamma _{5}=1.5$. To make a clear comparison, the purple curves represent $T_{I}(h)$ in the network topologies that maximize the total number of infected individuals. The connectivity matrices employed are $a_{ij}=0.2$ (resp. $b_{ij}=0.2$) if the route for the susceptible individuals (resp. infected individuals) from patch *j* to patch *i* exists. (First row) The optimal network topologies when susceptible and infected individuals can follow different network topologies. These topologies are given by two directed graphs. (Second row) The optimal network topology when the network is assumed equal for all types of individuals. Informally speaking, the manner of determining the best graph when $a_{ij}=b_{ij}$ is to study the sign of (11). Note that any path between patches 4 and 5 enhances the number of infected individuals. Figure A3 in the ESM describes the evolution of the total number of infected individuals in a network of 50 nodes

In summary, if the movement of all individuals is permitted but we can control the movement of the individuals depending on their status, the recommendation is to employ two directed graphs, one for the movement of the susceptible individuals and other one for the infected individuals; see Fig. 1 (first row).

To describe the topology that minimizes the total number of infected individuals when the movement of all individuals is permitted but there are no movement differences between them, i.e., $a_{ij}=b_{ij}$, we obtain the formula (see ESM)

10 $$\begin{aligned} T_{I}'(0)=\sum _{{\begin{array}{c} i,j=1\\ i\not =j \end{array}}}^{n}\frac{a_{ij}}{\beta _{j}} \left[ \left( \frac{\Delta _{j}}{\Delta _{i}}-1\right) +\frac{\mathcal {R}_{0,j}\mu }{\Delta _{j}} \left( \frac{\Gamma _{i}}{\Gamma _{j}}-1\right) \right] . \end{aligned}$$

As above, the contribution of the movement of individuals from patch *j* to patch *i* on the number of infected individuals is

11 $$\begin{aligned} \frac{a_{ij}}{\beta _{j}}\left[ \left( \frac{\Delta _{j}}{\Delta _{i}}-1\right) +\frac{\mathcal {R}_{0,j}\mu }{\Delta _{j}} \left( \frac{\Gamma _{i}}{\Gamma _{j}}-1\right) \right] . \end{aligned}$$

If $\Delta _{j}<\Delta _{i}$ and $\Gamma _{j}>\Gamma _{i}$, the movement of individuals in the route from patch *j* to patch *i* decreases the total number of infected individuals and the route in the opposite sense increases it. If $\Delta _{j}>\Delta _{i}$ and $\Gamma _{i}<\Gamma _{j}$, the movement of susceptible individuals in the route from patch *j* to patch *i* diminishes the total number of the infected individuals and the movement of the infected individuals enhances it. A gross manner to determine the resulting contribution is as follows: If $\mathcal {R}_{0,j}\approx 1$, the negative influence of the movement of the infected individuals on its own size dominates the positive influence of the movement of the susceptible individuals. If $\mathcal {R}_{0,j}$ is considerably greater than 1, the positive influence of the movement of the susceptible individuals dominates. Obviously, the dominating factors in the expressions of $\Gamma$ and $\Delta$ are the terms in the denominators. In many cases, it is not possible to determine the sign of (11) from inequalities of type $\Delta _{i}<\Delta _{j}$ and $\Gamma _{i}<\Gamma _{j}$. For instance, we give an example in Fig. 1 (second row) where $\Delta _{4}<\Delta _{5}$ and $\Gamma _{4}<\Gamma _{5}$ and the movement of individuals from patch 4 to patch 5 and vice versa enhances the total number of infected individuals.

#### The Disease is Endemic in the Patches $i=1,\ldots ,m$ and the Patches $i=m+1,\ldots ,n$ are Disease Free

In the ESM, we have obtained the formula

12 $$\begin{aligned} T_I'(0)= & {} \sum _{\begin{array}{c} i,j=1\\ i\not =j \end{array}}^{m}a_{ij}\frac{1}{\beta _{j}}\left( \frac{\Delta _{j}}{\Delta _{i}}-1\right) +\sum _{j=1}^{m}\sum _{i=m+1}^{n}a_{ij}\left( \frac{-1}{\beta _{j}}\right) +\sum _{i=1}^{m}\sum _{j=m+1}^{n}a_{ij}\frac{\lambda _{j}}{\mu \Delta _i} \nonumber \\&+\sum _{{\begin{array}{c} i,j=1i\not =j \end{array}}}^{m}b_{ij}\frac{\mu \mathcal {R}_{0,j}}{\beta _{j}\Delta _j}\left( \frac{\Gamma _{i}}{\Gamma _{j}}-1\right) \nonumber \\&+\sum _{i=m+1}^{n}\sum _{j=1}^{m}b_{ij}\frac{\mu }{\beta _{j}}\left( \frac{\mathcal {R}_{0,j}-1}{\Delta _i-\beta _{i}\frac{\lambda _{i}}{\mu }}-\frac{\mathcal {R}_{0,j}}{\Delta _j}\right) , \end{aligned}$$

where again $\Delta _{i}=\gamma _{i}+\mu$. The three first terms in (12) determine the contribution of the movement of the susceptible individuals on the total number of infected individuals. A noticeable fact is that the movement of susceptible individuals between two disease-free patches has a negligible influence on the total number of infected individuals, see Fig. A.1 in ESM.

**Fig. 2:**
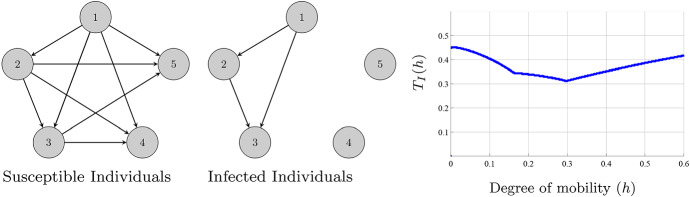
(Color figure online) Representation of the optimal topologies for the susceptible (left) and infected individuals (right). Representation of $T_{I}(h)$. The network is made of five nodes with parameters: $\lambda _{i}=0.5,\beta _i=1$ for all $i=1,\ldots ,5$, $\mu =0.5$,$\gamma _{1}=0.32$, $\gamma _{2}=0.3$, $\gamma _{3}=0.2$, $\gamma _{4}=0.6$ and $\gamma _{5}=0.51$. The connectivity matrices employed are $a_{ij}=0.1$ (resp. $b\_{ij}=0.1$) if the route for the susceptible individuals (resp. infected individuals) from patch *j* to patch *i* exists. The disease is endemic in the patches 1, 2 and 3, and it is eradicated in the patches 4 and 5. After simple computations, we obtain that $\Gamma _{1}>\Gamma _{2}>\Gamma _{3}$. Moreover, $\xi _{ij}>0$ for $i=4,5$ and $j=1,2,3$

The movement of susceptible individuals from a patch in which the disease is endemic to a disease-free patch always reduces the number of infected individuals, especially when the contact rate of the departing patch is small. In contrast, the movement of susceptible individuals from a disease-free patch to a patch in which the disease is endemic always increases the number of infected individuals. The influence of the movement of susceptible individuals between two patches where the disease is endemic follows the same rule as in (6). Hence, the topology for the susceptible individuals that minimizes the number of infected individuals is as follows: First, we construct all possible links from the patches in which the disease is endemic to the disease-free patches. Then, among the patches where the disease is endemic, we construct a directed graph with the arrows pointing to the patch with the largest value of $\Delta$; see Fig. 2.

The last two terms in (12) determine the contribution of the movement of the infected individuals on its own population size. The influence of the movement of the infected individuals between two patches where the disease is endemic is the same as in (6). On the other hand, the influence of the movement of the infected individuals from a patch where the disease is endemic (patch *j*) to a disease-free patch (patch *i*) is

$$\begin{aligned} \xi _{ij}=\frac{\mathcal {R}_{0,j}-1}{\Delta _{i}-\beta _{i}\frac{\lambda _{i}}{\mu }}-\frac{\mathcal {R}_{0,j}}{\Delta _{j}}. \end{aligned}$$

**Fig. 3:**
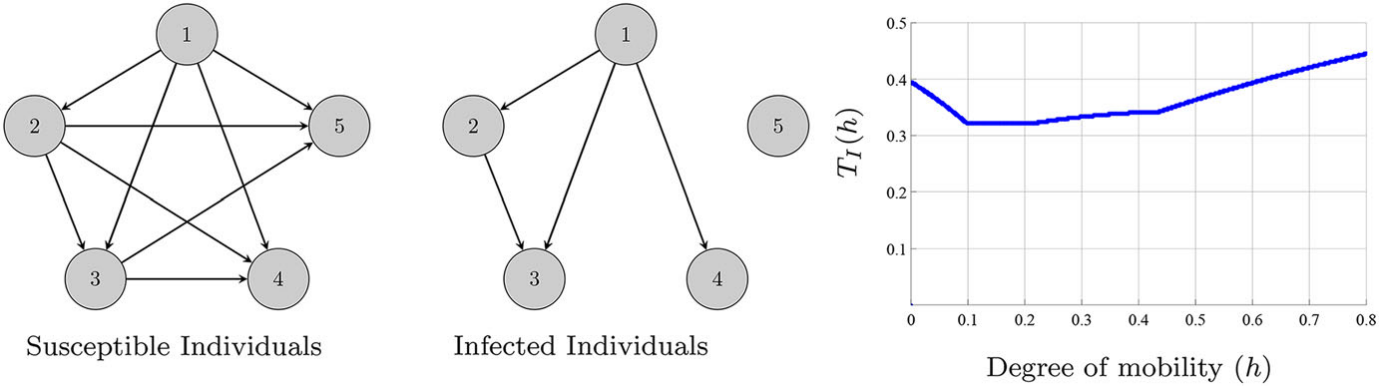
(Color figure online) Representation of the optimal topologies for the susceptible (left) and infected individuals (right). Representation of $T_{I}(h)$. The network is made of five nodes with the same value of parameters as in Fig. 2 except for $\gamma _1=0.4$. Again, the disease is endemic in the patches 1, 2 and 3, and it is eradicated in the patches 4 and 5 and $\Gamma _{1}>\Gamma _{2}>\Gamma _{3}$, but now $\xi _{41}<0$, which originates the new path from 1 to 4.

If $\xi _{ij}>0$ (resp. $<0$), the movement of the infected individuals has a detrimental (resp. beneficial) influence. If the basic reproduction number of the arriving patch is $\mathcal {R}_{0,i}<1$ but $\mathcal {R}_{0,i}\approx 1$ and the reproduction number of the leaving patch $\mathcal {R}_{0,j}$ is much larger than 1, then $\xi _{ij}>0$. This means that any small perturbative event could lead to $\mathcal {R}_{0,i}>1$ and could provoke the continuous presence of the epidemic. On the other hand, the movement of infected individuals from a disease-free patch to a patch in which the disease is endemic and the movement between two disease-free patches have a negligible influence on the number of infected individuals; see Fig. A 1 in ESM. To construct the topology for the infected individuals that minimizes its own population size, we have to follow the next steps: First, we construct all the links from the patches in which the disease is endemic to disease-free patches provided $\xi _{ij}<0$. Then, among the patches where the disease is endemic, we construct a directed graph with the arrows always pointing to the patch with the lowest value of $\Gamma$; see Fig. 2. We present an example with one of the values of $\xi _{ij}<0$ in Fig. 3.

### Optimal Topologies for Populations with a High Degree of Mobility

A possible manner to reduce the number of infected individuals is the isolation of the susceptible individuals in one patch and the exportation of the infected individuals to any of the other patches. If these movements are done almost instantaneously, i.e., $h\longrightarrow +\infty$, the number of infected individuals will go to zero, see the ESM and Fig. A2 therein. Thus, the optimal topologies are directed graphs both for the susceptible and infected individuals but each one pointing to different patches. Note that if the greatest value of $\gamma _{i}$ and the lowest value of $\Gamma _{i}$ are attained in different patches, then the recommended topologies made for small values of *h* are also valid for large values of *h*.

Despite this similarity, the influence of the network topology on the number of infected individuals for highly mobile populations is different from that with reduced mobility. For example, if the movement is symmetric, the topology and the precise values of the diffusion parameters $a_{ij}$, $b_{ij}$, and $c_{ij}$ do not have any influence on the number of infected individuals provided the topologies for the three groups are irreducible. In fact, if the disease is not completely eradicated,

13 $$\begin{aligned} \lim _{h\rightarrow +\infty }T_{I}(h)=n\left( \frac{(\sum _{i=1}^{n}\lambda _{i})(\sum _{i=1}^{n}\gamma _{i})(\sum _{i=1}^{n}\beta _{i})}{n\mu (n\mu +\sum _{i=1}^{n}\gamma _{i})}-n\mu \right) . \end{aligned}$$

**Fig. 4:**
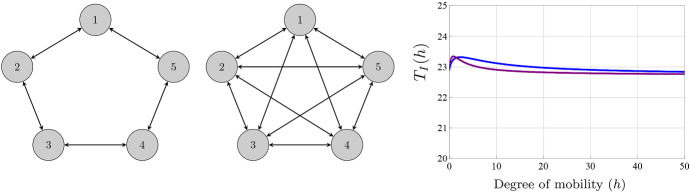
(Color figure online) (Left) Representation of two different networks of five nodes. (Right) Representation of $T_{I}(h)$. Model parameters: $\lambda _{i}=0.5$ and $\beta _{i}=0.1$ for $i=1,\ldots ,5$, $\mu =0.5$, $\gamma _{1}=0.3$, $\gamma _{2}=0.4$, $\gamma _{3}=0.5$, $\gamma _{4}=0.6$ and $\gamma _{5}=0.7$. We assume that $a_{ij}=b_{ij}$. The connectivity matrices are $a_{ij}=b_{ij}=0.1$ if the route exists and $a_{ij}=b_{ij}=0$ otherwise

We stress that (13) is independent of the presence of some patches where the disease is not endemic; see Fig. 4. The reader can consult (Arino 2009) for further results in this direction.

## Discussion

In this paper, we have described the topologies that minimize the number of infected individuals in epidemic models with spatially distributed populations. For simplicity, we have presented our results with the classical SIR model with patch structure (Allen et al. 2007; Arino 2009; Castillo-Chavez et al. 2016; Lamouroux et al. 2015). However, any epidemic model can be treated in an analogous manner, see ESM.

### The Movement Timescale is a Critical Factor for the Optimal Networks

For populations with reduced mobility, we assign a number to each topology so that two networks with the same number behave in the same manner, (see 6 and 12). With this strategy, the recommendation to reduce the total number of infected individuals is to employ two directed graphs, one for the susceptible individuals and other one for the infected individuals. As indicated in Shtilerman and Stone (2015), this type of graphs is frequently excluded because many tools in network theory assume irreducibility. The study for highly mobile populations requires a different methodology and new phenomena emerge. Inspired by Arino (2009), we have deduced that the conjunction of a symmetric movement and a high degree of mobility leads to perfect mixing, i.e., the population has the same behavior as a population that inhabits a single region with averaged values of the parameters. We stress that both elements are critical for the negligible influence of the spatial variables. For instance, the isolation of infected individuals in a unique region, i.e., employing two different directed graphs (one for the susceptible individuals and other for the infected individuals) pointing to different regions, always produces the global eradication of the disease for highly mobile populations; see Fig. A 2 in ESM. In this case, the movement is not symmetric and obviously there is no perfect mixing.

The computation of the basic reproduction number and the synchrony have been the dominant topics in spatial epidemiology (Allen et al. 2007; Johnstone-Robertson et al. 2020; Rohani et al. 1999; Sun 2016). Nevertheless, neither the computation of the basic reproduction numbers nor the synchrony are well understood in complex metapopulations. Moreover, the recommendations based on these results are sometimes rather restrictive. On the other hand, the next subsection shows that an analysis of the evolution of the total number of infected individuals can facilitate the control of the epidemic independently of the precise value of the basic reproduction number of the whole metapopulation.

### Theoretical Insights in Management

Next we provide several guidelines that complement recent results (Artzy-Randrup and Stone 2010; Heesterbeek 2015; Lamouroux et al. 2015; Silk 2019; Tien and Earn 2010): **1** The optimal topologies for populations with reduced mobility are suitable directed graphs as described in Sect. 3. The use of directed graphs normally yields to the concentration of infected individuals in one patch. Managers could take advantage of this concentration to eradicate the epidemic by means of an additional vaccination campaign in this patch.

**2** The patches with the highest contribution to reduce the number of infected individuals are those with endemic disease that are close to becoming disease free. The exportation of infected individuals from these patches is critical because any improvement of them could lead to the local eradication of the disease.

**3** For highly mobile populations with symmetric movement, the topology and the diffusion coefficients do not play any role on the number of infected individuals. Consequently, the suppression of connections between nodes has a negligible influence on the number of infected individuals provided the resulting network is irreducible and the movement is symmetric.

**4** A folkloric result in epidemiology claims that increasing the movement rates has a deleterious effect for disease eradication when there are patches with high risk of infection. Nevertheless, the analysis of Sect. 3.1.2 suggests that, under certain conditions, it is possible to see the opposite effect. Many authors have stressed that these paradoxical phenomena are similar to those in source sink theory (Lamouroux et al. 2015; Ruiz-Herrera and Torres 2020). These words deserve some caution. In metapopulation theory, for species with reduced mobility, if the movement from patch *i* to patch *j* has a positive (resp. negative) effect on the total population size, then the movement in the opposite sense has a negative (resp. positive) influence. However, as illustrated in Fig. 1 second row, it is possible to see the same influence on the total number of infected individuals for both routes in model (1).

### Future Research Directions

The generality of our methodology implies that our results do not depend on a particular model formulation and are applicable to a broad range of situations. Nevertheless, we have made several assumptions that would be desirable to drop in future works. For instance, model (1) assumes implicitly that individuals continually change the number of effective contacts per unit of time (Keeling and Eames 2005). A possible next step could be to adapt our results in pairwise models. On the other hand, we always assume that the dynamical behavior of (1) is simple, namely the global attraction to an equilibrium. However, the presence of oscillations and chaotic patterns is broadly documented in epidemiology (Barrientos et al. 2017; Ruiz-Herrera 2020). In this context, our results are not valid and a different approach is needed. Other important challenge is the analysis of populations with an intermediate degree of mobility, i.e., a finite value of *h* that is not close to zero. After profuse numerical computations, we have observed that $T_{I}(h)$ follows the same pattern (independently of the topology): $T_{I}(h)$ is essentially constant beyond a threshold $\widetilde{h}$. In the interval $(0,\widetilde{h})$, $T_{I}(h)$ can exhibit oscillatory behaviors but at most, either a local maximum or a local minimum. For instance, $T_{I}(h)$ is never of sinusoidal type.

## Supplementary Information

Below is the link to the electronic supplementary material.

Supplementary material 1 (pdf 2109 KB)
