# Supplementary material for: The Role of Movement Patterns in Epidemic Models on Complex Networks
Source: Bull Math Biol. 2021 Aug 19;83(10):98. doi: 10.1007/s11538-021-00929-w (PMC8376740; doi:10.1007/s11538-021-00929-w)
Supplement: Supplementary file 1 — Supplementary material 1 (pdf 2109 KB) [file 11538_2021_929_MOESM1_ESM.pdf]

Electronic Supplementary Material

Here we derive the results that we have used in the manuscript.

A The SIR model with spatial structure

A.1 Basic Dynamical Properties of the Model

Definition A.1. *We say that a square matrix $\mathcal{A} = (a_{ij})$ of order n is a connectivity matrix if*

$$\sum_{i=1}^{n} a_{ij} = 0 \tag{1.1}$$

for all $j = 1, ..., n$ with

$$a_{ii} \leq 0 \quad and \quad a_{ij} \geq 0 \tag{1.2}$$

for all $i, j = 1, ..., n$, $i \neq j$.

Given $\mathcal{A} = (a_{ij})$, $\mathcal{B} = (b_{ij})$ and $\mathcal{C} = (c_{ij})$ three connectivity matrices of order n, a classical SIR model with patch structure is

$$\begin{cases} S_i' &= \lambda_i - \mu S_i - \beta_i S_i I_i + \sum_{j=1}^{n} h a_{ij} S_j \\ I_i' &= \beta_i S_i I_i - (\gamma_i + \mu) I_i + \sum_{j=1}^{n} h b_{ij} I_j \\ R_i' &= \gamma_i I_i - \mu R_i + \sum_{j=1}^{n} h c_{ij} R_j \end{cases} \tag{1.3}$$

for all $i = 1, ..., n$ with $h \geq 0$. The dynamical behaviour in each patch depends on the basic reproduction number

$$\mathcal{R}_{0,i} = \frac{\beta_i \lambda_i}{\mu(\gamma_i + \mu)}.$$

Specifically, if $\mathcal{R}_{0,i} > 1$, the endemic equilibrium $\left(\frac{\gamma_i+\mu}{\beta_i}, \frac{\mu}{\beta_i}(\mathcal{R}_{0,i} - 1), \frac{\gamma_i}{\beta_i}(\mathcal{R}_{0,i} - 1)\right)$ is a global attractor for the system

$$\begin{cases} S_i' &= \lambda_i - \mu S_i - \beta_i S_i I_i \\ I_i' &= \beta_i S_i I_i - (\gamma_i + \mu) I_i \\ R_i' &= \gamma_i I_i - \mu R_i. \end{cases} \tag{1.4}$$

Otherwise, the disease free equilibrium $\left(\frac{\lambda_i}{\mu}, 0, 0\right)$ is a global attractor.
The total population size at time t in model (1.3) is

$$N(t) = \sum_{i=1}^{n} S_i(t) + I_i(t) + R_i(t).$$

Adding all the equations we have

$$N'(t) = \sum_{i=1}^{n} \lambda_i - \mu N(t)$$

Therefore,

$$\lim_{t\to\infty} N(t) = \frac{1}{\mu}\sum_{i=1}^{n} \lambda_i. \tag{1.5}$$

In the sequel we analyze the variation with respect to h of the total population size of the infected individuals at equilibrium. In mathematical terms, we study the function

$$T_I(h) = \sum_{i=1}^{n} I_i^*(h) \tag{1.6}$$

with

$$(S_1^*(h), I_1^*(h), R_1^*(h), ..., S_n^*(h), I_n^*(h), R_n^*(h))$$

the equilibrium of (1.3), which is a global attractor.

A.2 The disease is endemic in all patches, $\mathcal{R}_{0,i} > 1$ for $i = 1, ..., n$

Theorem A.1. *Fix all parameters in (1.3) except h. Assume that $\mathcal{R}_{0,i} > 1$ for $i = 1, ..., n$. Then*

$$T_I'(0) = \sum_{\substack{i,j=1\\ i\neq j}}^{n} \frac{a_{ij}}{\beta_j}\left(\frac{\gamma_j+\mu}{\gamma_i+\mu} - 1\right) + \sum_{\substack{i,j=1\\ i\neq j}}^{n} b_{ij}\frac{\mu}{\beta_j}\left(\frac{(\mathcal{R}_{0,j}-1)}{(\mathcal{R}_{0,i}-1)}\frac{\mathcal{R}_{0,i}}{(\gamma_i+\mu)} - \frac{\mathcal{R}_{0,j}}{(\gamma_j+\mu)}\right) \tag{1.7}$$

Proof. We know that $S_i^*(0) = \frac{\gamma_i+\mu}{\beta_i}$ and $I_i^*(0) = \frac{\mu}{\beta_i}(\mathcal{R}_{0,i} - 1)$ for all $i = 1, ..., n$. Moreover

$$0 = \lambda_i - \mu S_i^*(h) - \beta_i S_i^*(h) I_i^*(h) + h\left(\sum_{j=1}^{n} a_{ij} S_j^*(h)\right).$$

After deriving with respect to h and evaluating at $h = 0$, we obtain that

$$0 = -\mu {S_i^*}'(0) - \beta_i {S_i^*}'(0) I_i^*(0) - \beta_i S_i^*(0){I_i^*}'(0) + \sum_{j=1}^{n} a_{ij} S_j^*(0).$$

Using that $I_i^*(0) = \frac{\mu}{\beta_i}(\mathcal{R}_{0,i} - 1)$, we get

$$0 = -\mu\mathcal{R}_{0,i}{S_i^*}'(0) - \beta_i S_i^*(0){I_i^*}'(0) + \sum_{j=1}^{n} a_{ij} S_j^*(0).$$

Thus,

$${S_i^*}'(0) = \frac{-\beta_i S_i^*(0){I_i^*}'(0) + \sum_{j=1}^{n} a_{ij} S_j^*(0)}{\mu\mathcal{R}_{0,i}}. \tag{1.8}$$

On the other hand, we have that

$$0 = \beta_i S_i^*(h) I_i^*(h) - (\gamma_i + \mu) I_i^*(h) + h\left(\sum_{j=1}^{n} b_{ij} I_j^*(h)\right).$$

After deriving and evaluating at $h = 0$, the previous expression leads to

$$0 = \beta_i {S_i^*}'(0) I_i^*(0) + \beta_i S_i^*(0) {I_i^*}'(0) - (\gamma_i + \mu) {I_i^*}'(0) + (\sum_{j=1}^{n} b_{ij} I_j^*(0)).$$

Recalling that $S_i^*(0) = \frac{\gamma_i + \mu}{\beta_i}$, we deduce that

$$0 = \beta_i {S_i^*}'(0) I_i^*(0) + \sum_{j=1}^{n} b_{ij} I_j^*(0). \tag{1.9}$$

Inserting expression (1.8) into (1.9), we obtain that

$$0 = \beta_i \left(\frac{-\beta_i S_i^*(0) {I_i^*}'(0) + \sum_{j=1}^{n} a_{ij} S_j^*(0)}{\mu \mathcal{R}_{0,i}} \right) I_i^*(0) + \sum_{j=1}^{n} b_{ij} I_j^*(0)$$

Using that $S_i^*(0) = \frac{\gamma_i + \mu}{\beta_i}$ and $I_i^*(0) = \frac{\mu}{\beta_i}(\mathcal{R}_{0,i} - 1)$ for all $i = 1, ..., n$, we arrive at

$$\frac{(\gamma_i + \mu)(\mathcal{R}_{0,i} - 1)}{\mathcal{R}_{0,i}} {I_i^*}'(0) = \frac{\mathcal{R}_{0,i} - 1}{\mathcal{R}_{0,i}} \sum_{j=1}^{n} a_{ij} S_j^*(0) + \sum_{j=1}^{n} b_{ij} I_j^*(0).$$

We conclude that

$${I_i^*}'(0) = \frac{1}{\gamma_i + \mu} \left(\sum_{j=1}^{n} a_{ij} S_j^*(0) \right) + \frac{1}{\gamma_i + \mu} \frac{\mathcal{R}_{0,i}}{\mathcal{R}_{0,i} - 1} \sum_{j=1}^{n} b_{ij} I_j^*(0)$$

and so

$$T_I'(0) = \sum_{i,j=1}^{n} \frac{a_{ij} S_j^*(0)}{\gamma_i + \mu} + \sum_{i,j=1}^{n} b_{ij} \frac{\mathcal{R}_{0,i}}{\mathcal{R}_{0,i} - 1} \frac{1}{\gamma_i + \mu} I_j^*(0)$$

Next, we use condition (1.1) in the previous expression. Notice that

$$T_I'(0) = \sum_{\substack{i,j=1 \\ i \neq j}}^{n} \frac{a_{ij} S_j^*(0)}{\gamma_i + \mu} + \sum_{i=1}^{n} \frac{a_{ii} S_i^*(0)}{\gamma_i + \mu} + \sum_{\substack{i,j=1 \\ i \neq j}}^{n} b_{ij} \frac{\mathcal{R}_{0,i}}{\mathcal{R}_{0,i} - 1} \frac{1}{\gamma_i + \mu} I_j^*(0) + \sum_{i=1}^{n} b_{ii} \frac{\mathcal{R}_{0,i}}{\mathcal{R}_{0,i} - 1} \frac{1}{\gamma_i + \mu} I_i^*(0)$$

Therefore,

$$T_I'(0) = \sum_{\substack{i,j=1 \\ i \neq j}}^{n} \frac{a_{ij}}{\beta_j} \left(\frac{\gamma_j + \mu}{\gamma_i + \mu} - 1 \right) + \sum_{\substack{i,j=1 \\ i \neq j}}^{n} b_{ij} \frac{\mu}{\beta_j} \left(\frac{\mathcal{R}_{0,j} - 1}{(\mathcal{R}_{0,i} - 1)} \frac{\mathcal{R}_{0,i}}{(\gamma_i + \mu)} - \frac{\mathcal{R}_{0,j}}{(\gamma_j + \mu)} \right).$$

□

Remark A.1. *If $a_{ij} = b_{ij}$ for all $i, j = 1, ..., n$, then*

$$T_I'(0) = \sum_{\substack{i,j=1 \\ i \neq j}}^{n} \frac{a_{ij}}{\beta_j} \left(\left(\frac{\gamma_j + \mu}{\gamma_i + \mu} - 1 \right) + \mu \left(\frac{\mathcal{R}_{0,j} - 1}{(\mathcal{R}_{0,i} - 1)} \frac{\mathcal{R}_{0,i}}{(\gamma_i + \mu)} - \frac{\mathcal{R}_{0,j}}{(\gamma_j + \mu)} \right) \right)$$

A.3 The disease is endemic in the patches $i = 1, ..., m$ and the patches $i = m+1, ..., n$ are disease-free

Theorem A.2. *Fix all parameters in (1.3) except h. Assume that* $\mathcal{R}_{0,i} > 1$ *for* $i = 1, ..., m$ *and* $\mathcal{R}_{0,i} < 1$ *for* $i = m+1, ..., n$. *Then*

$$T_I'(0) = \sum_{\substack{i,j=1 \\ i\neq j}}^{m} a_{ij}\frac{1}{\beta_j}\left(\frac{\gamma_j+\mu}{\gamma_i+\mu}-1\right) + \sum_{j=1}^{m}\sum_{i=m+1}^{n} a_{ij}\left(\frac{-1}{\beta_j}\right) + \sum_{i=1}^{m}\sum_{j=m+1}^{n} a_{ij}\frac{\lambda_j}{\mu(\gamma_i+\mu)}$$

$$+ \sum_{\substack{i,j=1 \\ i\neq j}}^{m} b_{ij}\frac{\mu}{\beta_j}\left(\frac{\mathcal{R}_{0,j}-1}{(\mathcal{R}_{0,i}-1)}\frac{\mathcal{R}_{0,i}}{(\gamma_i+\mu)} - \frac{\mathcal{R}_{0,j}}{(\gamma_j+\mu)}\right)$$

$$+ \sum_{i=m+1}^{n}\sum_{j=1}^{m} b_{ij}\frac{\mu}{\beta_j}\left((\mathcal{R}_{0,j}-1)\frac{1}{\gamma_i+\mu-\beta_i\frac{\lambda_i}{\mu}} - \frac{\mathcal{R}_{0,j}}{\gamma_j+\mu}\right).$$

Proof. We know that $S_i^*(0) = \frac{\gamma_i+\mu}{\beta_i}$ and $I_i^*(0) = \frac{\mu}{\beta_i}(\mathcal{R}_{0,i}-1)$ for all $i = 1, ..., m$ and $S_i^*(0) = \frac{\lambda_i}{\mu}$ and $I_i^*(0) = 0$ for all $i = m+1, ..., n$. Moreover,

$$0 = \lambda_i - \mu S_i^*(h) - \beta_i S_i^*(h) I_i^*(h) + h\left(\sum_{j=1}^{n} a_{ij} S_j^*(h)\right).$$

After deriving and evaluating at $h = 0$, we obtain that

$$0 = -\mu {S_i^*}'(0) - \beta_i {S_i^*}'(0) I_i^*(0) - \beta_i S_i^*(0) {I_i^*}'(0) + \sum_{j=1}^{n} a_{ij} S_j^*(0)$$

for $i = 1, ..., m$ and

$$0 = -\mu {S_i^*}'(0) - \beta_i S_i^*(0) {I_i^*}'(0) + \sum_{j=1}^{n} a_{ij} S_j^*(0)$$

for $i = m+1, ..., n$. Thus,

$${I_i^*}'(0) = \frac{-\mu\mathcal{R}_{0,i}{S_i^*}'(0) + \sum_{j=1}^{n} a_{ij} S_j^*(0)}{\beta_i S_i^*(0)} \tag{1.10}$$

for $i = 1, ..., m$. On the other hand,

$$0 = \beta_i S_i^*(h) I_i^*(h) - (\gamma_i+\mu) I_i^*(h) + h\left(\sum_{j=1}^{n} b_{ij} I_j^*(h)\right).$$

After deriving and evaluating at $h = 0$, the previous expression leads to

$$0 = \beta_i {S_i^*}'(0) I_i^*(0) + \beta_i S_i^*(0) {I_i^*}'(0) - (\gamma_i+\mu) {I_i^*}'(0) + \sum_{j=1}^{m} b_{ij} I_j^*(0)$$

for $i = 1, ..., m$ and

$$0 = \beta_i S_i^*(0) {I_i^*}'(0) - (\gamma_i+\mu) {I_i^*}'(0) + \sum_{j=1}^{m} b_{ij} I_j^*(0)$$

for $i = m+1, ..., n$. We deduce that

$$S_i^{*\prime}(0) = \frac{-1}{\mu(\mathcal{R}_{0,i} - 1)} \sum_{j=1}^{m} b_{ij} I_j^*(0) \tag{1.11}$$

for $i = 1, ..., m$ and

$$0 = \left(\beta_i \frac{\lambda_i}{\mu} - (\gamma_i + \mu)\right) I_i^{*\prime}(0) + \sum_{j=1}^{m} b_{ij} I_j^*(0) \tag{1.12}$$

for $i = m+1, ..., n$. Inserting (1.11) into (1.10), we obtain that

$$I_i^{*\prime}(0) = \frac{1}{\gamma_i + \mu} \sum_{j=1}^{n} a_{ij} S_j^*(0) + \frac{\mathcal{R}_{0,i}}{(\mathcal{R}_{0,i} - 1)(\gamma_i + \mu)} \sum_{j=1}^{n} b_{ij} I_j^*(0)$$

for $i = 1, ..., m$. Next, using that $S_j^*(0) = \frac{\gamma_j + \mu}{\beta_j}$ and $I_j^*(0) = \frac{\mu}{\beta_j}(\mathcal{R}_{0,j} - 1)$ for $j = 1, ..., m$ and $S_j^*(0) = \frac{\lambda_j}{\mu}$ and $I_j^*(0) = 0$ for $j = m+1, ..., n$ in the previous expressions, we deduce that

$$I_i^{*\prime}(0) = \frac{1}{\gamma_i + \mu} \sum_{j=1}^{m} a_{ij} \frac{\gamma_j + \mu}{\beta_j} + \frac{1}{\gamma_i + \mu} \sum_{j=m+1}^{n} a_{ij} \frac{\lambda_j}{\mu} + \frac{\mathcal{R}_{0,i}}{(\mathcal{R}_{0,i} - 1)(\gamma_i + \mu)} \sum_{j=1}^{m} b_{ij} \frac{\mu}{\beta_j}(\mathcal{R}_{0,j} - 1)$$

for $i = 1, ..., m$ and

$$I_i^{*\prime}(0) = \sum_{j=1}^{m} b_{ij} \frac{\mu}{\beta_j}(\mathcal{R}_{0,j} - 1) \frac{1}{\gamma_i + \mu - \beta_i \frac{\gamma_i}{\mu}}$$

for $i = m+1, ..., n$. Finally, using (1.1) and (1.2), we arrive to the given formula.

A.4 Triangular Connections as Optimal configurations for $h \longrightarrow +\infty$

Let us consider a connectivity matrix $\mathcal{A} = (a_{ij})$ for the susceptible individuals such that there is a connection from patch i to j if and only if $i > j$. This implies that $\mathcal{A}$ is an upper triangular matrix with $a_{11} = 0$. On the reverse, the connectivity matrix $\mathcal{B} = (b_{ij})$ for the infected population is taken such that there is a connection from patch i to j if and only if $i < j$. Thus, $\mathcal{B}$ is a lower triangular matrix with $b_{nn} = 0$. In other words, we are considering directed graphs pointing to the region 1 and n for the susceptible individuals and the infected individuals respectively.

Since the total population size is bounded, the equilibrium

$$(S_1^*(h), I_1^*(h), R_1^*(h), ..., S_n^*(h), I_n^*(h), R_n^*(h))$$

of model (1.3) is bounded for all $h \geq 0$ and satisfies

$$\left\{ \begin{array}{lll} 0 & = & \frac{1}{h}(\lambda_i - \mu S_i^*(h) - \beta_i S_i^*(h) I_i^*(h)) + \sum_{j=1}^n a_{ij} S_j^*(h) \\ 0 & = & \frac{1}{h}(\beta_i S_i^*(h) I_i^*(h) - (\gamma_i + \mu) I_i^*(h)) + \sum_{j=1}^n b_{ij} I_j^*(h) \\ 0 & = & \frac{1}{h}(\gamma_i I_i^*(h) - \mu R_i^*(h)) + \sum_{j=1}^n c_{ij} R_j^*(h) \end{array} \right.$$

for all $i = 1, ..., n$. Making $h \longrightarrow \infty$ in the previous expressions, we obtain that

$$\left\{ \begin{array}{lll} 0 & = & \sum_{j=1}^n a_{ij} S_j^*(\infty) \\ 0 & = & \sum_{j=1}^n b_{ij} I_j^*(\infty) \\ 0 & = & \sum_{j=1}^n c_{ij} R_j^*(\infty) \end{array} \right.$$

for all $i = 1, ..., n$ with

$$\lim_{h \longrightarrow \infty} (S_1^*(h), I_1^*(h), R_1^*(h), ..., S_n^*(h), I_n^*(h), R_n^*(h)) = (S_1^*(\infty), I_1^*(\infty), R_1^*(\infty), ..., S_n^*(\infty), I_n^*(\infty), R_n^*(\infty)).$$

For the vectors

$$S^*(\infty) = (S_1^*(\infty)..., S_n^*(\infty)),$$
$$I^*(\infty) = (I_1^*(\infty)..., I_n^*(\infty)),$$

this simply means that $S^*(\infty) \in Ker\mathcal{A}$ and $I^*(\infty) \in Ker\mathcal{B}$. Having said that, the choice of the connectivity matrices as triangular matrices implies that $S_i^*(\infty) = 0$ for $i = 2, \ldots, n$, while $I_i^*(\infty) = 0$ for $i = 1, \ldots, n-1$. Now, by adding the first n equations of system (1.3), we obtain easily that

$$S_1^*(\infty) = \frac{1}{\mu} \sum_{i=1}^{n} \lambda_i.$$

Note that this limit leads to $I_n^*(\infty) = 0$, as a consequence of (1.5).

Remark A.2. *The previous argument also works if $\mathcal{A}$ and $\mathcal{B}$ are matrices associated with directed architectures pointing to patches s_1 and s_2 with $s_1 \neq s_2$.*

A.5 Symmetric Movement and $h \longrightarrow \infty$

Arguing as above, $(S_1^*(\infty), I_1^*(\infty), R_1^*(\infty), ..., S_n^*(\infty), I_n^*(\infty), R_n^*(\infty)) \in Ker\mathcal{P}$ with

$$\mathcal{P} = \begin{pmatrix} \mathcal{A} & 0 & 0 \\ 0 & \mathcal{B} & 0 \\ 0 & 0 & \mathcal{C} \end{pmatrix}$$

a $3n \times 3n$ square matrix where $\mathcal{A} = (a_{ij})$, $\mathcal{B} = (b_{ij})$ and $\mathcal{C} = (c_{ij})$ are the $n \times n$ square matrices associated with (1.3). Using that these matrices are symmetric and satisfy (1.2), we know that

$$Ker\mathcal{P} = \{\sigma_1 v_1 + \sigma_2 v_2 + \sigma_3 v_3 : \sigma_i \in \mathbb{R}\}$$

with $v_1 = (1, ..., 1, 0, ..., 0, 0, ..., 0)$, $v_2 = (0, ..., 0, 1, ..., 1, 0, ..., 0)$, and $v_3 = (0, ..., 0, 0, ..., 0, 1, ..., 1)$. This implies that $S_i^*(\infty) = \sigma_1$, $I_i^*(\infty) = \sigma_2$ and $R_i^*(\infty) = \sigma_3$ for all $i = 1, ..., n$ for suitable constants $\sigma_i \in [0, \infty)$. Next we determine these constants:
Summing the equations of (1.3), we obtain that

$$\begin{cases} \sum_{i=1}^n \lambda_i & = & n\mu\sigma_1 + (\sum_{i=1}^n \beta_i)\sigma_1\sigma_2 \\ (\sum_{i=1}^n \beta_i)\sigma_1\sigma_2 & = & (n\mu + \sum_{i=1}^n \gamma_i)\sigma_2 \\ (\sum_{i=1}^n \gamma_i)\sigma_2 & = & (n\mu)\sigma_3 \end{cases} \tag{1.13}$$

After simple manipulations, we deduce that if $\sigma_2 > 0$, then

$$\sigma_1 = \frac{1}{\sum_{i=1}^n \beta_i}(n\mu + \sum_{i=1}^{n} \gamma_i)$$

and

$$\sigma_2 = \frac{\sum_{i=1}^n \lambda_i \sum_{i=1}^n \beta_i - (n\mu + \sum_{i=1}^n \gamma_i)n\mu}{(n\mu + \sum_{i=1}^n \gamma_i) \sum_{i=1}^n \beta_i}.$$

If $\sigma_2 = 0$, then

$$\sigma_1 = \frac{1}{n\mu} \sum_{i=1}^{n} \lambda_i, \qquad \sigma_2 = 0.$$

An important fact is that the obtained values do not depend on the coefficients of the connectivity matrices, but do depend on the number of patches.

A.6 Supplementary Figures

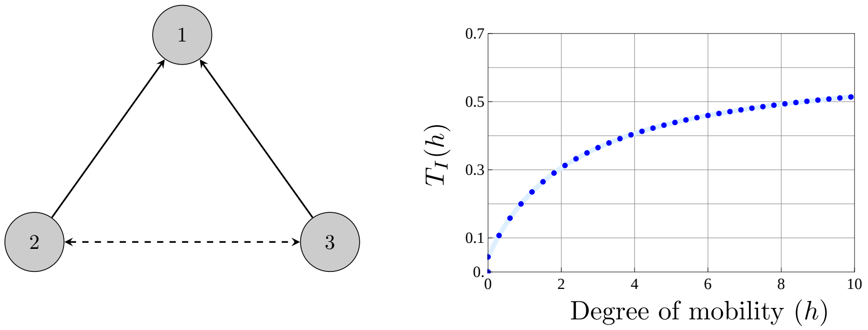

Figure A.1: (Left) Representation of a network with three nodes where the disease is endemic in patch 1 and it is eradicated in patches 2 and 3. (Right) Representation of $T_I(h)$ for $h \in (0, 10)$. Model parameters: $\lambda_i = 0.5$ for $i = 1, 2, 3$, $\mu = 0.5$, $\beta_1 = 2$, $\beta_2 = 0.7$, $\beta_3 = 0.6$, $\gamma_1 = 1.2$, $\gamma_2 = 1 = \gamma_3$. We assume that $a_{ij} = b_{ij}$. The connectivity matrices in the continuous curve and in the dashed curve are $a_{ij} = b_{ij} = 0.2$ if the route exists and $a_{ij} = b_{ij} = 0$ otherwise. The negligible influence of the movement of individuals between the patches 2 and 3 is clearly visible.

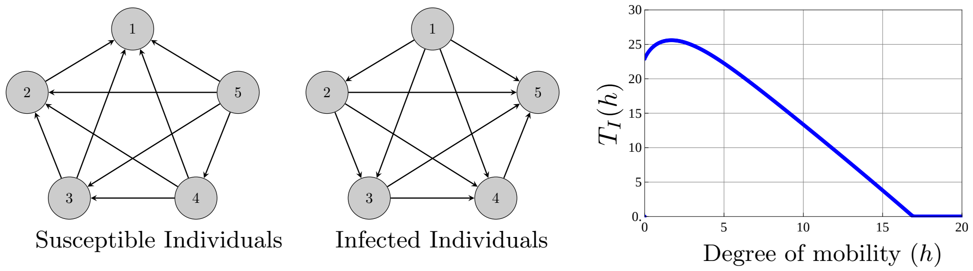

Figure A.2: (Left) Representation of the optimal networks. (Right) Representation of $T_I(h)$. Model parameters: $\lambda_i = 0.5$ and $\beta_i = 0.1$ for $i = 1, ..., 5$, $\mu = 0.5$, $\gamma_1 = 0.3$, $\gamma_2 = 0.4$, $\gamma_3 = 0.5$, $\gamma_4 = 0.6$ and $\gamma_5 = 0.7$. The connectivity matrices employed are $a_{ij} = 0.2$ (resp. $b_{ij} = 0.2$) if the route for the susceptible individuals (resp. infected individuals) from patch j to patch i exists. The total eradication of the disease is observed for values of $h > 17$.

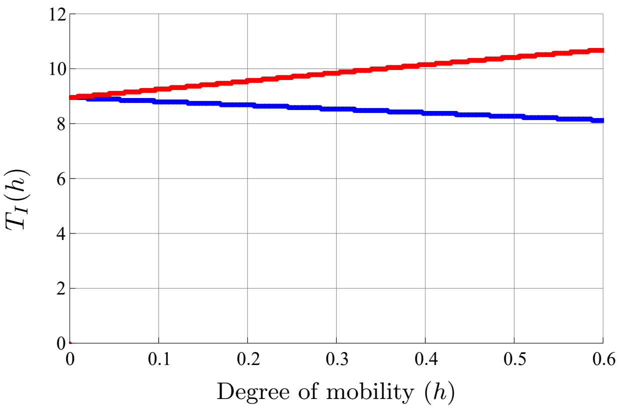

Figure A.3: Representation of $T_I(h)$ for a network of 50 nodes. Model parameters: $\lambda_i = 5$ and $\beta_i = 7$ for all i, $\mu = 1$, $\gamma_i = 5 + i/2$ for $i = 1, \ldots, 50$. The connectivity matrices employed are $a_{ij} = 1/50$ (resp. $b_{ij} = 1/50$) if the route for the susceptible individuals (resp. infected individuals) from patch j to patch i exists. The blue curve corresponds to the optimal topologies for the susceptible and infected individuals according to Section 3.1.1 of the main text. The purple curve corresponds to the network the maximize the total number of infected individuals.

B The SI model with patch structure

B.1 Basic Dynamical Properties of the Model

Given $\mathcal{A} = (a_{ij})$ and $\mathcal{B} = (b_{ij})$ two connectivity matrices of order n, we consider the model

$$\begin{cases} S_i' &= \lambda_i - \mu S_i - \beta_i S_i I_i + \sum_{j=1}^n h a_{ij} S_j \\ I_i' &= \beta_i S_i I_i - \mu I_i + \sum_{j=1}^n h b_{ij} I_j \end{cases} \tag{2.14}$$

for all $i = 1, ..., n$ with $h \geq 0$. All the parameters in (2.14) are positive. From a biological point of view, the main difference between (2.14) and (1.3) is that the disease is chronic. In other words, the infected individuals never change their status. The basic reproduction number in the patch i is

$$\mathcal{R}_{0,i} = \frac{\beta_i \lambda_i}{\mu^2}.$$

If $\mathcal{R}_{0,i} > 1$, the endemic equilibrium $\left(\frac{\mu}{\beta_i}, \frac{\mu}{\beta_i}(\mathcal{R}_{0,i} - 1)\right)$ is a global attractor for the system

$$\begin{cases} S_i' &= \lambda_i - \mu S_i - \beta_i S_i I_i \\ I_i' &= \beta_i S_i I_i - \mu I_i. \end{cases} \tag{2.15}$$

Otherwise, the disease free equilibrium $\left(\frac{\lambda_i}{\mu}, 0\right)$ is a global attractor.

Arguing as in the previous section, see (1.5), the total population size at time t in model (2.14)

$$N(t) = \sum_{i=1}^n S_i(t) + I_i(t)$$

satisfies that

$$\lim_{t \to +\infty} N(t) = \frac{1}{\mu} \sum_{i=1}^n \lambda_i. \tag{2.16}$$

In the sequel we analyze the variations with respect to h of the total population size of the infected individuals at equilibrium. Specifically, we study the function

$$T_I(h) = \sum_{i=1}^{n} I_i(h) \tag{2.17}$$

with

$$(S_1(h), I_1(h), ..., S_n(h), I_n(h))$$

the equilibrium of (2.14), which is a global attractor.

B.2 The disease is endemic in all patches, $\mathcal{R}_{0,i} > 1$ for $i = 1, ..., n$

Theorem B.1. *Fix all parameters in (2.14) except h. Assume that $\mathcal{R}_{0,i} > 1$ for $i = 1, ..., n$. Then*

$$T_I'(0) = \sum_{\substack{i,j=1 \\ i \neq j}}^{n} b_{ij} \frac{1}{\beta_j} \left(\frac{\mathcal{R}_{0,j} - 1}{\mathcal{R}_{0,i} - 1} \mathcal{R}_{0,i} - \mathcal{R}_{0,j} \right) \tag{2.18}$$

Proof. We know that $S_i(0) = \frac{\mu}{\beta_i}$ and $I_i(0) = \frac{\mu}{\beta_i}(\mathcal{R}_{0,i} - 1)$ for all $i = 1, ..., n$. Moreover

$$0 = \lambda_i - \mu S_i(h) - \beta_i S_i(h) I_i(h) + h \left(\sum_{j=1}^{n} a_{ij} S_j(h) \right).$$

After deriving and evaluating at $h = 0$, we obtain that

$$0 = -\mu S_i'(0) - \beta_i S_i'(0) I_i(0) - \beta_i S_i(0) I_i'(0) + \sum_{j=1}^{n} a_{ij} S_j(0).$$

Using that $S_i(0) = \frac{\mu}{\beta_i}$ and $I_i(0) = \frac{\mu}{\beta_i}(\mathcal{R}_{0,i} - 1)$, we get

$$I_i'(0) = -\mathcal{R}_{0,i} S_i'(0) + \sum_{j=1}^{n} a_{ij} \frac{1}{\beta_j}. \tag{2.19}$$

On the other hand, we have that

$$0 = \beta_i S_i(h) I_i(h) - \mu I_i(h) + h \left(\sum_{j=1}^{n} b_{ij} I_j(h) \right).$$

After deriving and evaluating at $h = 0$, the previous expression leads to

$$S_i'(0) = -\frac{1}{\mathcal{R}_{0,i} - 1} \left(\sum_{j=1}^{n} b_{ij} \frac{\mathcal{R}_{0,j} - 1}{\beta_j} \right).$$

Inserting these expression into (2.19), we obtain that

$$I_i'(0) = \frac{\mathcal{R}_{0,i}}{\mathcal{R}_{0,i} - 1} \left(\sum_{j=1}^{n} b_{ij} \frac{\mathcal{R}_{0,j} - 1}{\beta_j} \right) + \sum_{j=1}^{n} a_{ij} \frac{1}{\beta_j}.$$

We deduce that

$$T_I'(0) = \sum_{i,j=1}^{n} b_{ij} \frac{\mathcal{R}_{0,i}}{\mathcal{R}_{0,i}-1} \left(\frac{\mathcal{R}_{0,j}-1}{\beta_j} \right) + \sum_{i,j=1}^{n} a_{ij} \frac{1}{\beta_j}.$$

Finally, using (1.1) in the previous expression, we conclude

$$T_I'(0) = \sum_{\substack{i,j=1 \\ i \neq j}}^{n} b_{ij} \frac{1}{\beta_j} \left(\frac{\mathcal{R}_{0,i}(\mathcal{R}_{0,j}-1)}{(\mathcal{R}_{0,i}-1)} - \mathcal{R}_{0,j} \right).$$

B.3 The disease is endemic in the regions i for $i = 1, ..., m$ and it is eradicated in the regions $i = m+1, ..., n$

We know that $S_i(0) = \frac{\mu}{\beta_i}$ and $I_i(0) = \frac{\mu}{\beta_i}(\mathcal{R}_{0,i}-1)$ for all $i = 1, ..., m$ and $S_i(0) = \frac{\lambda_i}{\mu}$ and $I_i(0) = 0$ for all $i = m+1, ..., n$. Moreover,

$$0 = \lambda_i - \mu S_i(h) - \beta_i S_i(h) I_i(h) + h \left(\sum_{j=1}^{n} a_{ij} S_j(h) \right)$$

for $i = 1, ..., m$. After deriving and evaluating at $h = 0$, we obtain that

$$0 = \mu S_i'(0) - \beta_i S_i'(0) I_i(0) - \beta_i S_i(0) I_i'(0) + \sum_{j=1}^{n} a_{ij} S_j(0)$$

for $i = 1, ..., m$. Thus,

$$I_i'(0) = -\mathcal{R}_{0,i} S_i'(0) + \sum_{j=1}^{m} a_{ij} \frac{1}{\beta_j} + \sum_{j=m+1}^{n} a_{ij} \frac{\lambda}{\mu^2} \tag{2.20}$$

for $i = 1, ..., m$. On the other hand, we have that

$$0 = \beta_i S_i(h) I_i(h) - \mu I_i(h) + h \left(\sum_{j=1}^{n} b_{ij} I_j(h) \right)$$

for all $i = 1, ..., n$. After deriving and evaluating at $h = 0$, the previous expression leads to

$$0 = \beta_i S_i'(0) I_i(0) + \beta_i S_i(0) I_i'(0) - \mu I_i'(0) + \sum_{j=1}^{m} b_{ij} I_j(0)$$

for $i = 1, ..., m$ and

$$0 = \beta_i S_i(0) I_i'(0) - \mu I_i'(0) + \sum_{j=1}^{m} b_{ij} I_j(0)$$

for $i = m+1, ..., n$. We deduce that

$$0 = \beta_i S_i'(0) I_i(0) + \sum_{j=1}^{m} b_{ij} I_j(0) \tag{2.21}$$

for $i = 1, ..., m$ and

$$0 = \left(\beta_i \frac{\lambda_i}{\mu} - \mu\right) I_i'(0) + \sum_{j=1}^{m} b_{ij} I_j(0) \tag{2.22}$$

for $i = m+1, ..., n$. Therefore,

$$S_i'(0) = -\sum_{j=1}^{n} b_{ij} \frac{\mathcal{R}_{0,j} - 1}{\mathcal{R}_{0,i} - 1}$$

for $i = 1, ..., m$ and

$$I_i'(0) = \sum_{j=1}^{m} b_{ij} \frac{\mu(\mathcal{R}_{0,j} - 1)}{\beta_j \left(\beta_i \frac{\lambda_i}{\mu} - \mu\right)}$$

for $i = m+1, ..., n$. Using (2.20), we arrive at

$$I_i'(0) = \mathcal{R}_{0,i} \left(\sum_{j=1}^{n} b_{ij} \frac{\mathcal{R}_{0,j} - 1}{\mathcal{R}_{0,i} - 1} \right) + \sum_{j=1}^{m} a_{ij} \frac{1}{\beta_j} + \sum_{j=m+1}^{n} a_{ij} \frac{\lambda}{\mu^2}$$

for $i = 1, ..., m$. Arguing as in the previous subsection, we obtain the following formula

$$\begin{aligned} T_I'(0) = &\sum_{i=1}^{m} \sum_{j=m+1}^{n} a_{ji} \left(\frac{-1}{\beta_i}\right) + \sum_{i=1}^{m} \sum_{j=m+1}^{n} a_{ij} \frac{\lambda_j}{\mu^2} \\ &+ \sum_{\substack{i,j=1 \\ i \neq j}}^{m} b_{ij} \frac{1}{\beta_j} \left(\frac{\mathcal{R}_{0,j} - 1}{(\mathcal{R}_{0,i} - 1)} \mathcal{R}_{0,i} - \mathcal{R}_{0,j} \right) + \sum_{i=1}^{m} \sum_{j=m+1}^{n} -b_{ji} \frac{\mathcal{R}_{0,i}}{\beta_i} \\ &+ \sum_{i=m+1}^{n} \sum_{j=1}^{m} b_{ij} \frac{1}{\beta_j} \left(\mu(\mathcal{R}_{0,j} - 1) \frac{1}{\mu - \beta_i \frac{\lambda_i}{\mu}} - \mathcal{R}_{0,j} \right). \end{aligned}$$

B.4 Analysis for $h \longrightarrow \infty$

We omit the details of the proofs because the treatment for $h \longrightarrow \infty$ is exactly the same as that made in subsections 1.4 and 1.5.
